# Supplementary material for: The Interaction of HMGB1 with the Proinflammatory TREM-1 Receptor Generates Cytotoxic Lymphocytes Active against HLA-Negative Tumor Cells
Source: Int J Mol Sci. 2024 Jan 3;25(1):627. doi: 10.3390/ijms25010627 (PMC10779375; doi:10.3390/ijms25010627)
Supplement: Supplementary file 1 [file ijms-25-00627-s001.zip › ijms-2743922-supplementary.pdf]

Supplemental figures.

Supplemental Figure S1.

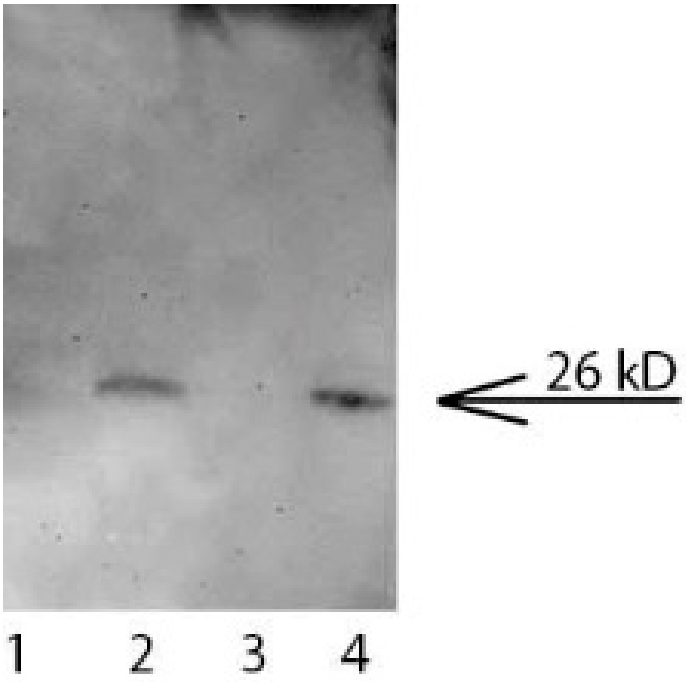

Control Western blot analysis experiment. Staining by specific antibodies to HMGB1:

1- TREM-1 protein (10µg)
2- HMGB1 protein (1 µg)
3- HMGB1 was incubated with monocytes in the presence of a BS^3 crosslinking agent. The cell lysate was purified by magnetic separation with conjugated nonspecific IgG.
4- HMGB1 was incubated with monocytes (no BS^3 crosslinking agent added). The cell lysate was purified by magnetic separation and specific antibodies against TREM-1.

Supplemental Figure S2.

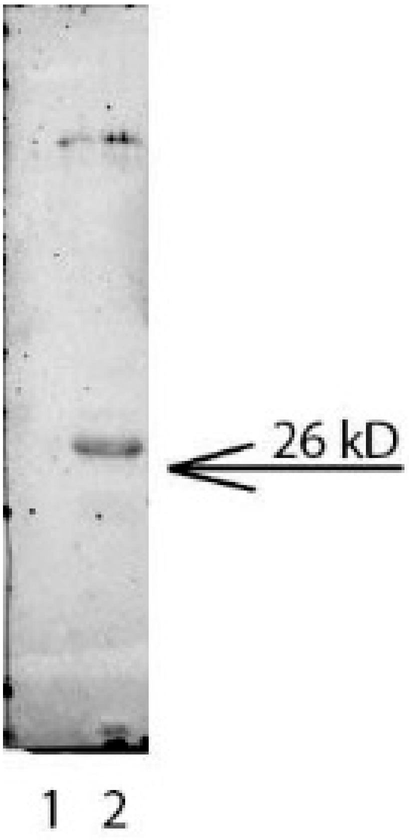

Control Western blot analysis experiment. Staining by specific antibodies to TREM-1:

1- HMGB1 protein (10 µg)
2- TREM-1 protein (10 µg)

Supplemental Figure S3.

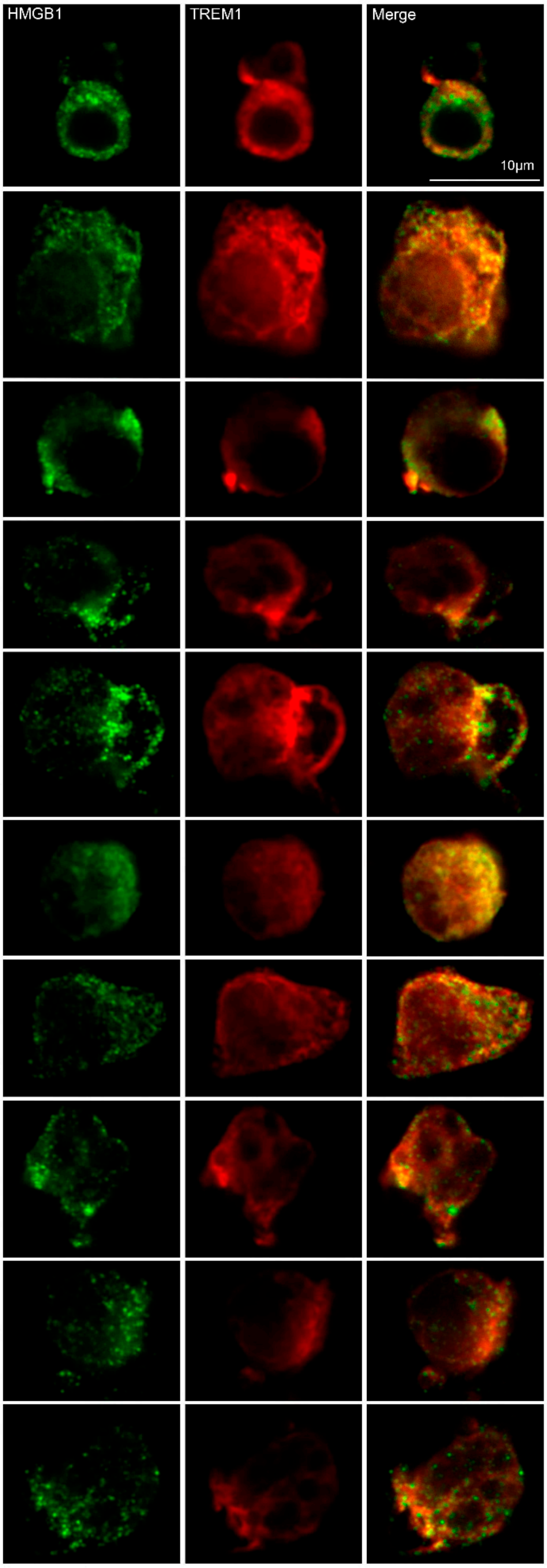

Collection of monocytes cells, stained by HMGB1(green) and TREM-1(red) and layers superposition (c).

Supplemental Figure S4.

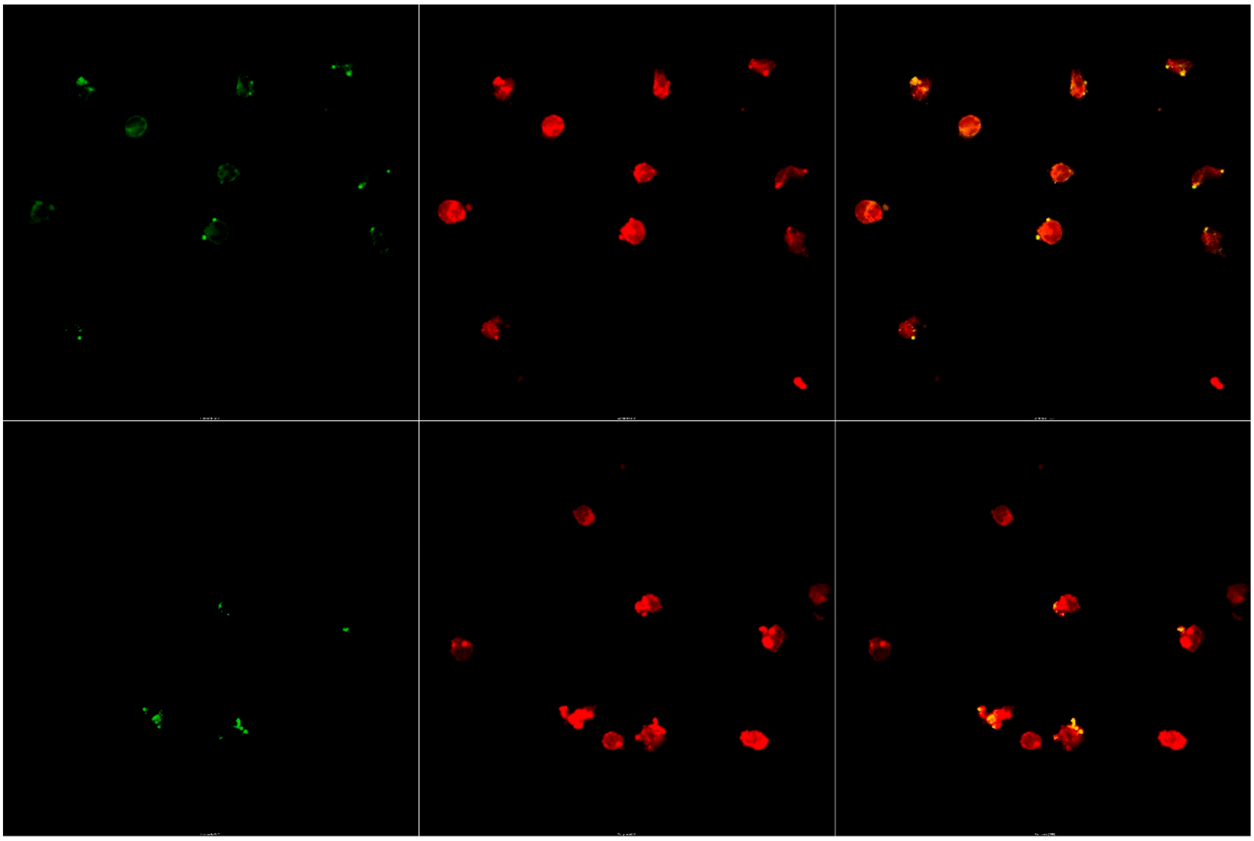

Typical confocal photos of HMGB1(green) and TREM-1(red) and layers superposition on the surface of monocytes.

Supplemental Figure S5.

A.

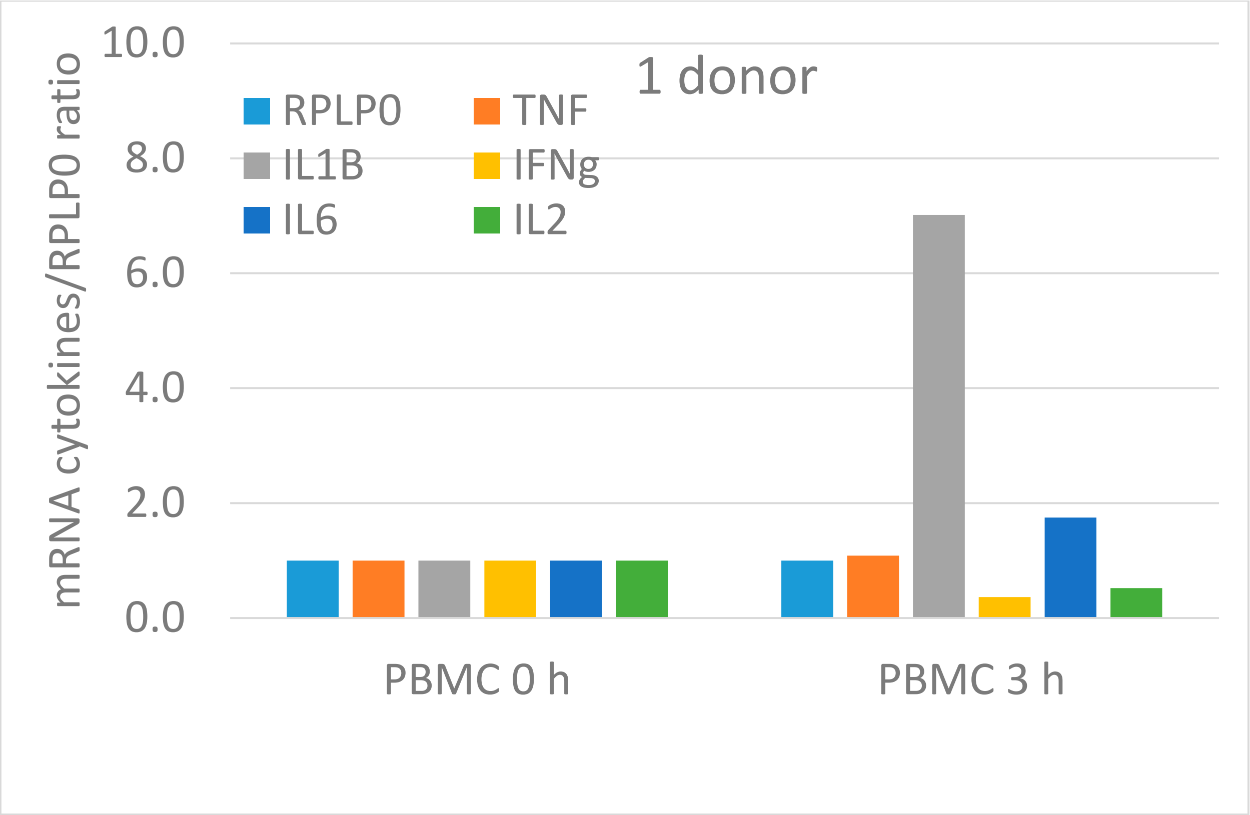

B.

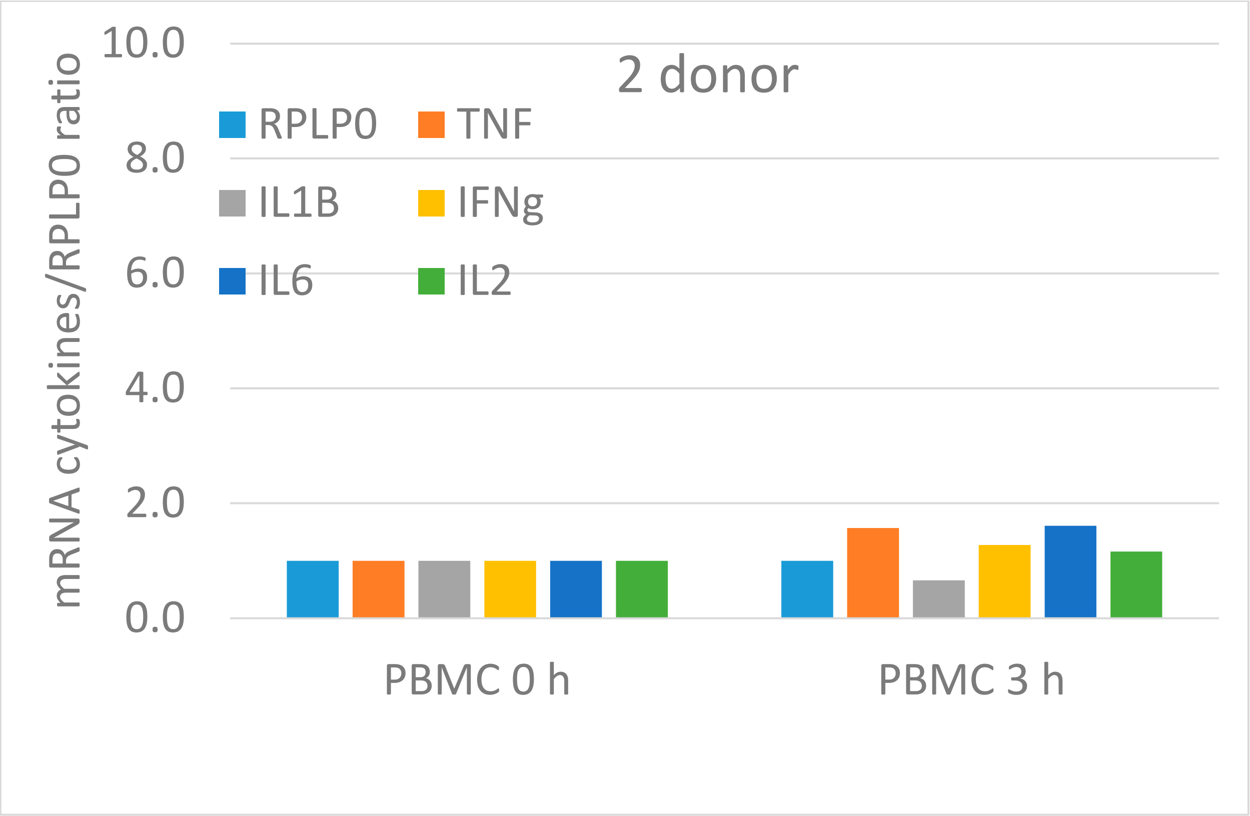

C.

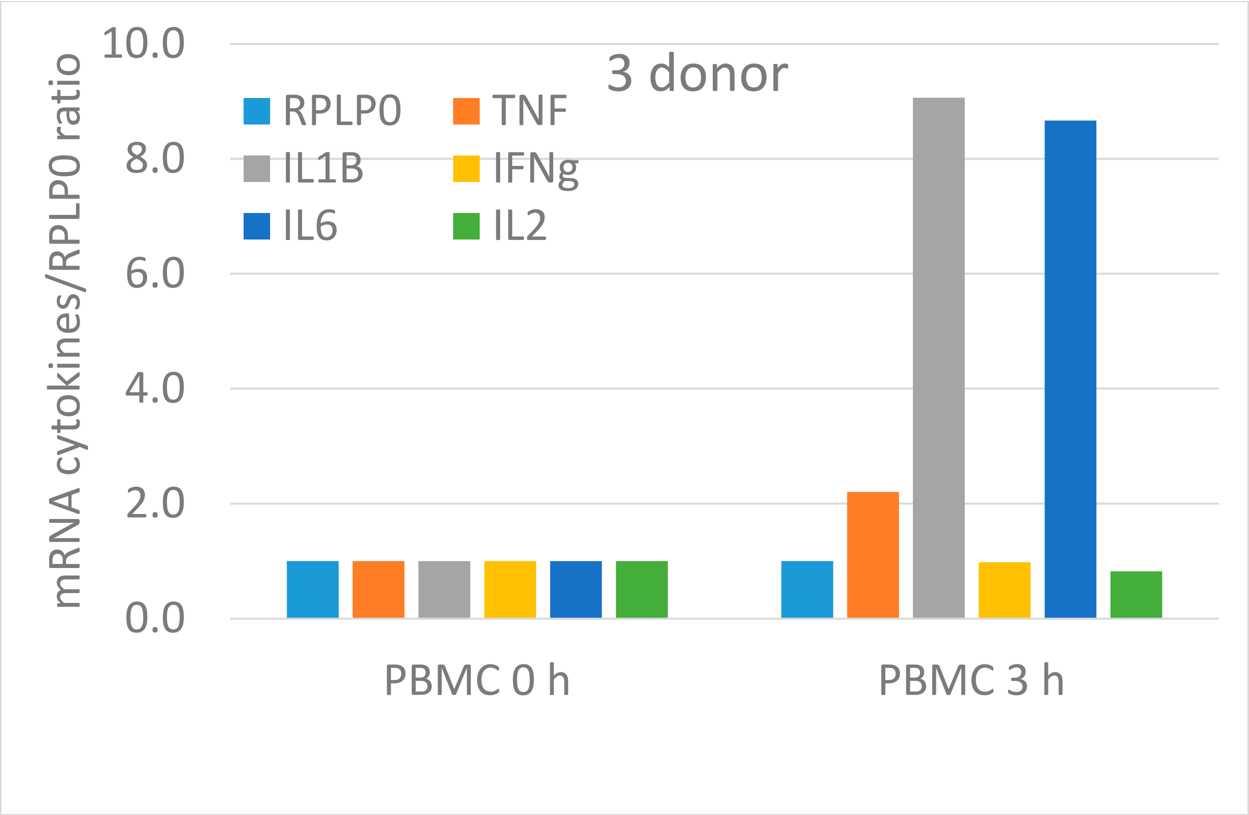

D.

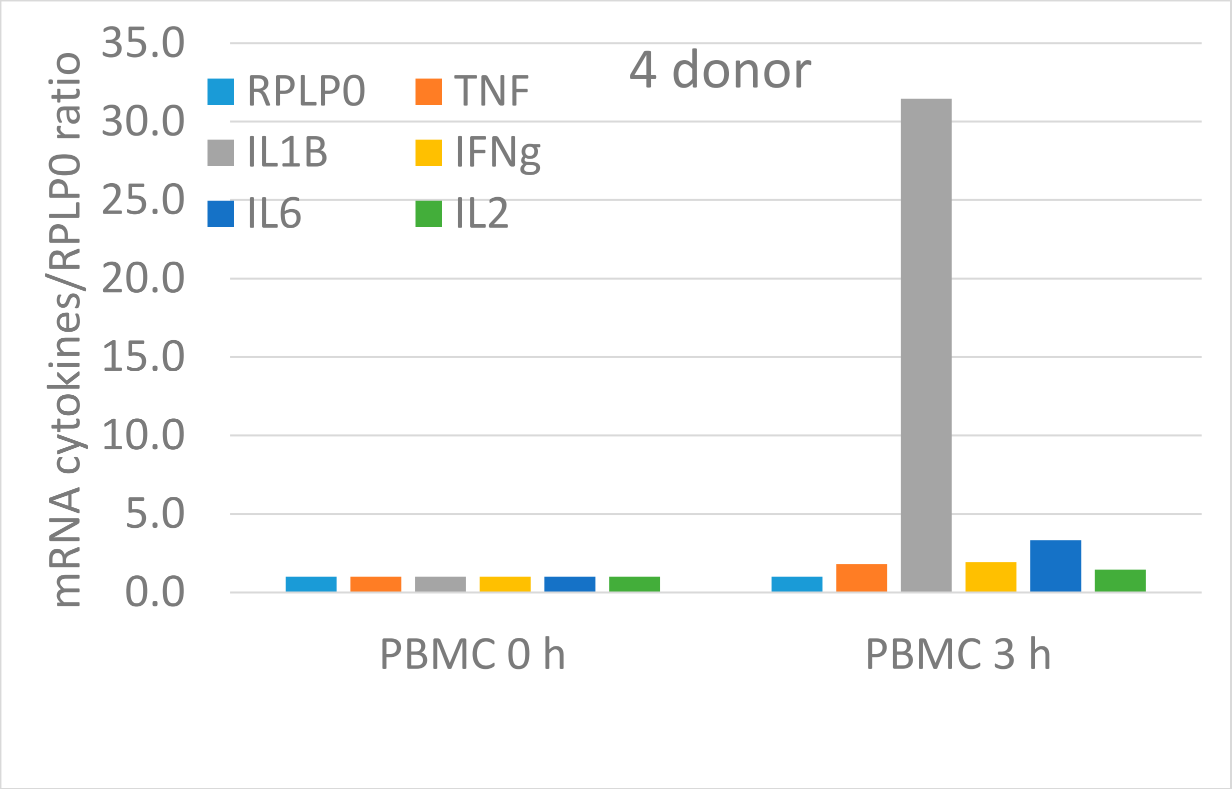

Activation of cytokines genes TNF, IL1B, IFNg, IL6, IL-2 in PBMC at zero point and after 3 hours of cultivation in RPMI-1640 medium without addition of any protein. PBMC from four different donors are used (A-D) to collect the samples for qPCR analysis. RPLP0 mRNA was used for samples normalization.

Supplemental Figure S6.

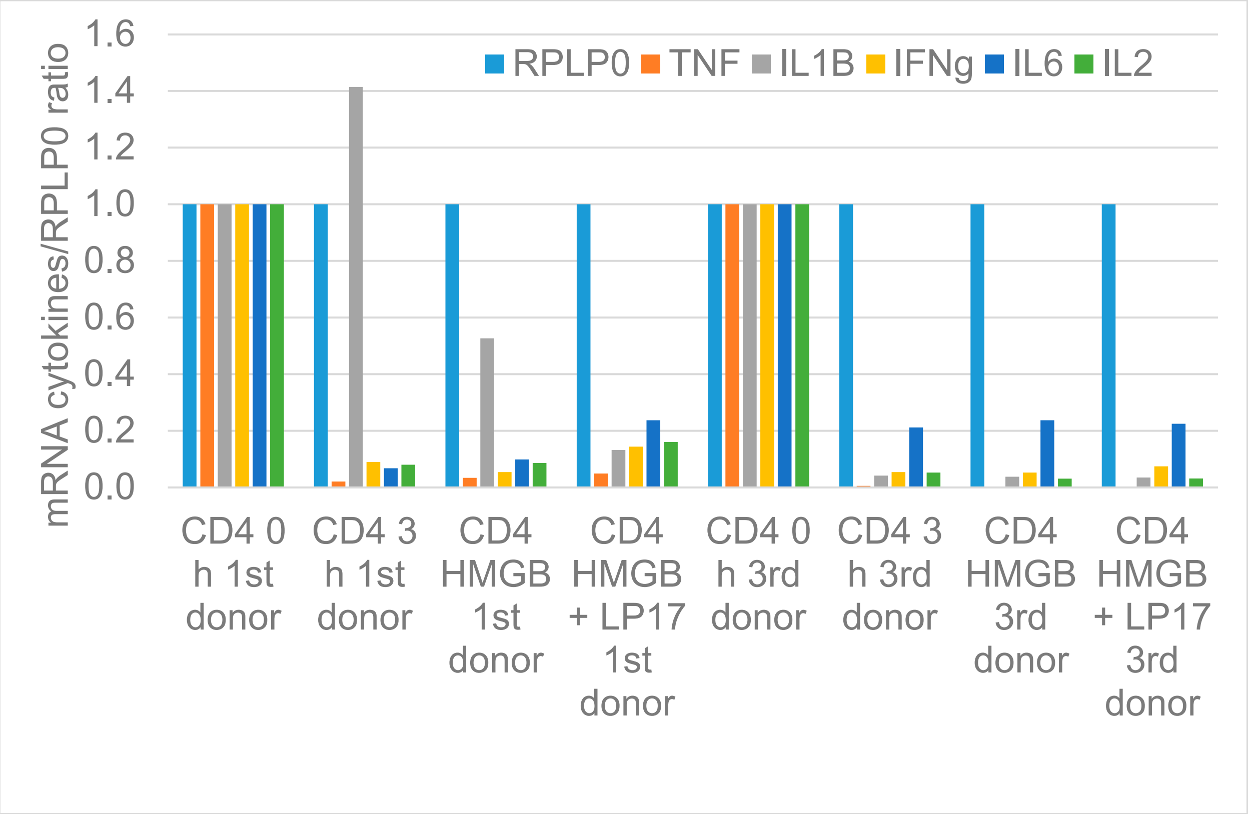

Activation of cytokines genes TNF, IL1B, IFNg, IL6, IL-2 in CD4+ T lymhpocyes. Cells from two different donors are presented, marked (1st donor) and (3rd donor). mRNA was measured on 0 hour and on 3 hour (0h and 3h) in presence of HMGB1 protein (HMGB) alone and HMGB1 + LP17 inhibitory peptide(HMGB+LP17). RPLP0 mRNA was used for samples normalization.
